# Affinity purified anti-citrullinated protein/peptide antibodies target antigens expressed in the rheumatoid joint

**DOI:** 10.1186/ar4683

**Published:** 2014-08-12

**Authors:** Elena Ossipova, Cátia Fernandes Cerqueira, Evan Reed, Nastya Kharlamova, Lena Israelsson, Rikard Holmdahl, Kutty Selva Nandakumar, Marianne Engström, Ulrike Harre, Georg Schett, Anca I Catrina, Vivianne Malmström, Yngve Sommarin, Lars Klareskog, Per-Johan Jakobsson, Karin Lundberg

**Affiliations:** Rheumatology Unit, Department of Medicine, Karolinska Institutet, CMM L8:04, Karolinska University Hospital Solna, 171 76 Stockholm, Sweden; Department of Medical Biochemistry and Biophysics, Karolinska Institutet, Scheeles väg 2, Karolinska Institutet, 171 77 Stockholm, Sweden; Department of Internal Medicine 3, University of Erlangen-Nuremberg, Ulmenweg 18, 91054 Erlangen, Germany; Euro-Diagnostica AB, Euro Diagnostica AB, P.O. Box 50117, 202 11 Malmö, Sweden; Rheumatology Unit, Department of Medicine, Karolinska Institutet, Karolinska University Hospital Solna, CMM L8:04, Karolinska University Hospital Solna, 171 76 Stockholm, Sweden

## Abstract

**Introduction:**

A major subset of patients with rheumatoid arthritis (RA) is characterized by the presence of circulating autoantibodies directed to citrullinated proteins/peptides (ACPAs). These autoantibodies, which are commonly detected by using an enzyme-linked immunosorbent assay (ELISA) based on synthetic cyclic citrullinated peptides (CCPs), predict clinical onset and a destructive disease course. In the present study, we have used plasma and synovial fluids from patients with RA, for the affinity purification and characterization of anti-CCP2 reactive antibodies, with an aim to generate molecular tools that can be used *in vitro* and *in vivo* for future investigations into the pathobiology of the ACPA response. Specifically, this study aims to demonstrate that the surrogate marker CCP2 can capture ACPAs that bind to autoantigens expressed *in vivo* in the major inflammatory lesions of RA (that is, in the rheumatoid joint).

**Methods:**

Plasma (n = 16) and synovial fluid (n = 26) samples were collected from RA patients with anti-CCP2 IgG levels of above 300 AU/mL. Total IgG was isolated on Protein G columns and subsequently applied to CCP2 affinity columns. Purified anti-CCP2 IgG was analyzed for reactivity and specificity by using the CCPlus® ELISA, in-house peptide ELISAs, Western blot, and immunohisto-/immunocytochemistry.

**Results:**

Approximately 2% of the total IgG pool in both plasma and synovial fluid was CCP2-reactive. Purified anti-CCP2 reactive antibodies from different patients showed differences in binding to CCP2 and differences in binding to citrullinated peptides from α-enolase, vimentin, fibrinogen, and collagen type II, illustrating different ACPA fine-specificity profiles. Furthermore, the purified ACPA bound not only *in vitro* citrullinated proteins but, more importantly, *in vivo*-generated epitopes on synovial fluid cells and synovial tissues from patients with RA.

**Conclusions:**

We have isolated ACPAs from plasma and synovial fluid and demonstrated that the CCP2 peptides, frequently used in diagnostic ELISAs, *de facto* act as surrogate antigens for at least four different, well-characterized, largely non-cross-reactive, ACPA fine specificities. Moreover, we have determined the concentration and proportion of CCP2-reactive IgG molecules in rheumatoid plasma and synovial fluid, and we have shown that the purified ACPAs can be used to detect both *in vitro*- and *in vivo*-generated citrullinated epitopes by various techniques. We anticipate that these antibodies will provide us with new opportunities to investigate the potential pathogenic effects of human ACPAs.

## Introduction

Autoimmunity in a major subset of patients with rheumatoid arthritis (RA) is characterized by the presence of disease-specific autoantibodies directed against post-translationally citrullinated proteins/peptides (ACPAs) [1–3]. These antibodies can be detected years before clinical manifestations of arthritis [4, 5], their presence predicts a more erosive disease course [6], and an enrichment in the joints compared with the circulation [7] suggests a local production and a pathogenic involvement. However, relatively little is known about the potential pathogenic effects of the human ACPA response. Hence, more in-depth molecular studies of ACPAs are warranted. To facilitate such future investigations, we have in the present study isolated and characterized ACPAs from RA synovial fluid (SF) and plasma.

Routine testing for ACPAs in clinical practice is commonly performed by using a commercial enzyme-linked immunosorbent assay (ELISA) based on cyclic citrullinated peptides (CCPs), the so-called “second generation” anti-CCP ELISA [2]. Today, this assay constitutes an important diagnostic tool for RA and is also widely used in research studies on ACPAs. However, anti-CCP2 reactive antibodies target synthetic peptides that do not correspond to any human protein sequence, and thus these antibodies act as surrogate markers for autoimmunity in RA without formally defining any reactivity against autoantigens present *in vivo*.

We and others have investigated the fine specificity of the ACPA response [8–12] and demonstrated reactivity to citrullinated epitopes on proteins expressed in the rheumatoid joint, including α-enolase [13, 14], fibrinogen [15], vimentin [16], collagen type II (CII) [17], immunoglobulin binding protein (BiP) [18], annexin [19], and histone 4 [20]. The frequencies of ACPAs reactive with single autoantigen-derived peptides are in most patient cohorts lower than the frequency of anti-CCP2 reactive antibodies, whereas the number of RA patients who are positive for any of these ACPA fine specificities is similar to the number of anti-CCP2-positive patients (typically around 60% to 70%) [21]. Notably, reactivity to the autoantigen-derived citrullinated peptides can also be detected in a minor subset of anti-CCP2-negative patients. Peptide-absorption experiments demonstrate limited cross-reactivity between different ACPA fine specificities, and different ACPA-reactivity patterns can be seen in different patients [10–12, 21].

Whether the same antibodies that react with the CCP2 peptides used in the diagnostic ELISA also react with citrullinated antigens expressed in tissues and cells from rheumatoid joints, including the four most extensively studied ACPA targets (citrullinated α-enolase, vimentin, fibrinogen, and collagen type II), has never been formally demonstrated (with the exception of citrullinated fibrinogen) [22]. A prerequisite for such studies involves the isolation of human anti-CCP2 reactive antibodies. Hence, in the present study, where we have affinity-purified ACPAs on CCP2 columns, we have had the opportunity to investigate the reactivity of anti-CCP2 IgG in detail. We have also been able to determine the concentration and proportion of CCP2-reactive IgG molecules in rheumatoid plasma and SF; importantly, we have demonstrated that the purified ACPAs can be used as molecular tools for the detection of *in vitro*- and *in vivo*-generated citrullinated epitopes by ELISA, Western blot, and immunohisto-/immunocytochemistry.

## Materials and methods

### Patients

Non-paired synovial fluid (n = 26) and plasma (n = 16) samples were collected with informed consent from RA patients attending the rheumatology clinic at Karolinska University Hospital, Stockholm, Sweden, from 2001 to 2011. Synovial fluid was collected from patients requiring arthrocentesis. All patients fulfilled the American College of Rheumatology/European League Against Rheumatism criteria for RA [23–25] and were selected on the basis of having high anti-CCP2 antibody levels (>300 AU/mL). Samples were stored at −20°C (short term) or −80°C (long term) until processed. The study was approved by the regional ethics committee at Karolinska Institutet.

### Affinity purification of human anti-CCP2 IgG

**Figure 1:**
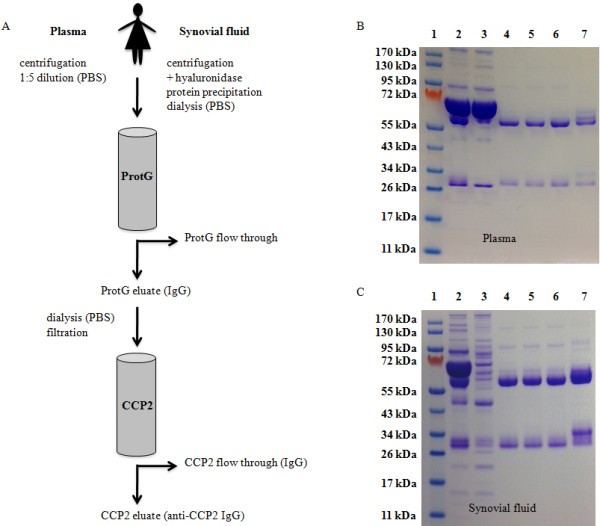
**Anti-CCP2 IgG was efficiently purified from plasma and synovial fluid.** Schematic illustration of the anti-CCP2 IgG purification process. **(A)** Sodium dodecyl sulphate-polyacrylamide gel electrophoresis (SDS-PAGE) followed by Coomassie staining of different protein fractions collected during the anti-CCP2 IgG purification process is shown. Representative samples from plasma **(B)** and synovial fluid **(C)**: molecular marker (lane 1); plasma/precipitated synovial fluid proteins (lane 2); protein G column flow through (lane 3); protein G column IgG eluate, before dialysis and filtration (lane 4); protein G column IgG eluate, after dialysis and filtration (lane 5); CCP2 column IgG flow through (lane 6); CCP2 column anti-CCP2 IgG eluate (lane 7). Lanes 2 and 3: 15 μg/well. Lanes 4 to 7: 5 μg/well. IgG, immunoglobulin G; PBS, phosphate-buffered saline.

Synovial fluid (10 to 20 mL per sample) was centrifuged at 4,000 rpm before supernatants were treated with hyaluronidase (Sigma-Aldrich, St. Louis, MO, USA) for 1 hour at 4°C in order to decrease viscosity. Proteins were precipitated by using saturated ammonium sulphate in accordance with standard protocol [26], dissolved in phosphate-buffered saline (PBS), and further dialyzed against PBS. Plasma (15 to 25 mL per sample) was centrifuged and diluted 1:5 (vol/vol) in PBS. IgGs from plasma and SF were purified on HiTrap Protein G HP columns (GE Healthcare, Stockholm, Sweden) in accordance with the instructions of the manufacturer. Eluted IgG fractions were dialyzed against PBS and filtered before being applied to the CCP2 affinity column, kindly provided by Euro-Diagnostica AB (Malmö, Sweden). The CCP2 column was subsequently washed with 10 column volumes of PBS before bound anti-CCP2 IgG was eluted by using 0.1 M glycine-HCl buffer (pH 2.7) and directly neutralized with 1 M Tris (pH 9) (Figure 1). Buffer exchange to PBS and concentration of antibodies was performed in one step, applying the 10-kDa Microsep™ UF Centrifugal Device (Pall Life Science, Port Washington, NY, USA) in accordance with the instructions of the manufacturer.

### CCP2 column

CCP2 peptides (2 mg) (corresponding to the patent-protected peptides used in the commercially available Immunoscan CCPlus® assay from Euro-Diagnostica AB) were coupled to 1 mL HiTrap columns (GE Healthcare). Recommended flow rate and backpressure specifications were followed (flow rate: 1 mL/min, maximum backpressure: 0.3 MPa). Performance of the columns was typically around 1 mg anti-CCP2 IgG/column.

### Calculations of total IgG and anti-CCP2 IgG concentrations in plasma and SF

Protein concentrations were determined in Protein G column eluate fractions (before and after dialysis and filtration) and in CCP2 column flow-through and eluate fractions by using the Bradford DC assay (Bio-Rad Laboratories, Hercules, CA, USA) and the Nanodrop Spectrophotometer (Thermo Scientific, Waltham, MA, USA) in parallel in accordance with the instructions of the manufacturers. The concentration (in milligrams per milliliter) of total IgG was calculated on the basis of the initial plasma or SF volume applied to the Protein G column and the amount of IgG eluted from the Protein G column (that is, before dialysis and filtration). The proportion (percentage) of anti-CCP2 IgG (of total IgG) was calculated on the basis of the amount of IgG applied to the CCP2 column (that is, after dialysis and filtration) and the amount of anti-CCP2 IgG eluted from the CCP2 column. The concentration (in milligrams per milliliter) of anti-CCP2 IgG was then calculated on the basis of this proportion (percentage) in relation to the concentration (in milligrams per milliliter) of total IgG in plasma and SF.

### SDS-PAGE

Recovery and purity of total IgG and anti-CCP2 IgG were analyzed by Coomassie brilliant blue staining after protein separation on sodium dodecyl sulphate-polyacrylamide gel electrophoresis (SDS-PAGE). In brief, samples were diluted in lithium dodecyl sulphate (LDS) sample buffer (Invitrogen, Carlsbad, CA, USA) containing dithiothreitol (DTT) and subjected to denaturation and reduction at 70°C for 10 minutes. Plasma, precipitated SF proteins, Protein G column flow-through fractions (at 15 μg/well), Protein G column eluate fractions (before and after dialysis and filtration), and CCP2 column flow-through and eluate fractions (at 5 μg/well) were loaded onto NuPAGE® Bis-Tris 4%-12% gels (Invitrogen) and run in 2-(*N*-morpholino)ethanesulfonic acid (MES)-SDS antioxidant-containing running buffer at 200 V (constant) for 45 minutes. Protein bands were visualized by Coomassie brilliant blue staining (0.2% Comassie blue, 7.5% glacial acetic acid, 50% methanol) in accordance with standard protocol.

### ELISAs

Anti-CCP2 reactivity was determined in 10 plasma and 11 SF samples and in the corresponding fractions collected during the anti-CCP2 IgG purification process (as described above). Samples were analyzed in duplicates and in serial dilutions (7 × 1:3 dilution steps) starting at 1:100 (plasma and SF) or at 10 μg/mL (fractions) by using the Immunoscan CCPlus® assay (Euro-Diagnostica AB) in accordance with the instructions of the manufacturer. Cutoff for positivity was 25 AU/mL. Reactivities to citrullinated peptides from α-enolase (CEP-1; amino acid 5-21), vimentin (Cit-vim; amino acid 60-75), fibrinogen (Cit-fib; amino acid 36-52), and collagen type II (Cit-C1; amino acid 359-369) were assayed in the same samples at the same dilutions by using in-house ELISAs as previously described [21]. Cutoff for positivity was calculated on the basis of the 98th percentile among 150 healthy controls and set to 10 AU/mL for all ACPA fine specificities.

### Western blot

Purified human fibrinogen (Merck, Darmstadt, Germany) depleted of immunoglobulins, recombinant human α-enolase, and recombinant mutated human vimentin were citrullinated *in vitro* by using rabbit skeletal muscle peptidylarginine deiminase 2 (PAD2) (Sigma-Aldrich) as previously described [27]. Citrullinated and uncitrullinated proteins (1 μg/well) were separated on NuPAGE® Bis-Tris 10% gels (Bio-Rad Laboratories), stained with Coomassie brilliant blue, or transferred to nitrocellulose membranes. Membranes were blocked with 5% milk, tris-buffered saline (TBS)/0.05% Tween, probed with a pool of 11 purified anti-CCP2 IgG eluate fractions from SF, or the corresponding CCP2 column flow-through IgG pool, at 2 μg/mL, for 1 hour at room temperature (RT), before being washed in PBS/0.05% Tween and incubated with horseradish peroxidase (HRP)-conjugated goat anti-human IgG (The Jackson Laboratory, Bar Harbor, ME, USA), diluted 1:20,000, for 1 hour at RT. After a final wash, membranes were subjected to enhanced chemiluminescence (ECL) development.

### Immunohisto-/immunocytochemistry

Synovial hip or knee biopsy specimens were obtained from three patients with RA by joint replacement surgery and were snap-frozen in dry ice-cool isopentane; serial cryostat sections (7 μm) were fixed with 2% (vol/vol) formaldehyde and stored at −80°C before being stained. Fresh SF mononuclear cells from three patients with RA were prepared by ficoll separation and fixed in 2% formaldehyde before being stained. The presence of citrullinated epitopes was detected by using a pool of 26 purified anti-CCP2 IgG eluate fractions from SF and the corresponding flow-through IgG pool. The antibody pools were biotinylated (Lightning-Link™ Biotin conjugation kit; Innova Biosciences, Cambridge, UK) and used at 10 μg/mL for staining synovial tissue or at 5 μg/mL for staining SF cells. PBS/saponine was used to permeabilize the cells. The vectastain detection system (ABC-elite kit, Vector Laboratories, Burlingame, CA, USA) was used for the tissue, and Streptavidin/HRP (DakoCytomation, Glostrup, Denmark) was used for the cells.

### Statistical analyses

Differences in antibody levels (in arbitrary units per milliliter) between different ACPA fine specificities in plasma and SF and in the purified anti-CCP IgG2 eluate fractions were examined by using Mann-Whitney *U* test for independent groups.

## Results

### Efficient purification of anti-CCP2 IgG on CCP2 affinity columns

Anti-CCP2 IgG was efficiently purified from 16 plasma and 26 SF samples by using Protein G and CCP2 affinity columns (Figure 1A). Fractions collected during the purification process were analyzed by Coomassie staining after separation on SDS-PAGE (Figure 1B and C). IgG isolation on the protein G column was confirmed to efficiently remove non-IgG materials. No degradation, and only minor loss of protein content (<6%), was observed during the dialysis and filtration step (data not shown). The CCP2 affinity columns demonstrated high capacity regarding the amount of target-protein loaded, and no destructive processes were detected during the elution, yielding pure and intact anti-CCP2 IgG with high recovery. The multiple bands around the molecular weight of the IgG heavy and light chains, seen mainly in the CCP2 eluate fractions from both plasma and SF, may represent different isoforms and modifications (for example, glycosylation [28]) of the IgG molecules.

### Concentration and proportion of anti-CCP2 IgG in plasma and synovial fluid

By measuring the protein concentration at every step of the purification process, we have calculated the concentration of anti-CCP2 IgG in the circulation and locally in the joint (Table 1). The concentration of anti-CCP2 IgG was higher in plasma (median value of 0.2 mg/mL) than in SF (median value of 0.06 mg/mL), but since the concentration of total IgG was also higher in plasma (median value of 14.6 mg/mL) compared with SF (median value of 3.5 mg/mL), in line with what has previously been described [29, 30], the proportion of IgG molecules with CCP2 reactivity was in fact marginally higher in SF (median value of 2.2%, with four samples above 6%) than in plasma (median value of 1.5% and a highest recorded proportion of 3.6%).

**Table 1:** **Concentrations and proportion of anti-CCP2 IgG in plasma and synovial fluid**

	Plasma (n = 16)		SF (n = 26)	
	Median	Range	Median	Range
Total IgG concentration, mg/mL	14.6	6.5-25.7	3.5	0.9-15.2
Anti-CCP IgG concentration, mg/mL	0.2	0.05-0.5	0.06	0.01-0.2
Proportion of anti-CCP IgG, %^a^	1.5	0.4-3.6	2.2	0.1-15.6

^a^Calculated as the percentage of anti-CCP2 IgG of total IgG. CCP, cyclic citrullinated peptide; IgG, immunoglobulin G; SF, synovial fluid.

### Anti-CCP2 IgG contain ACPA of various fine specificities

**Figure 2:**
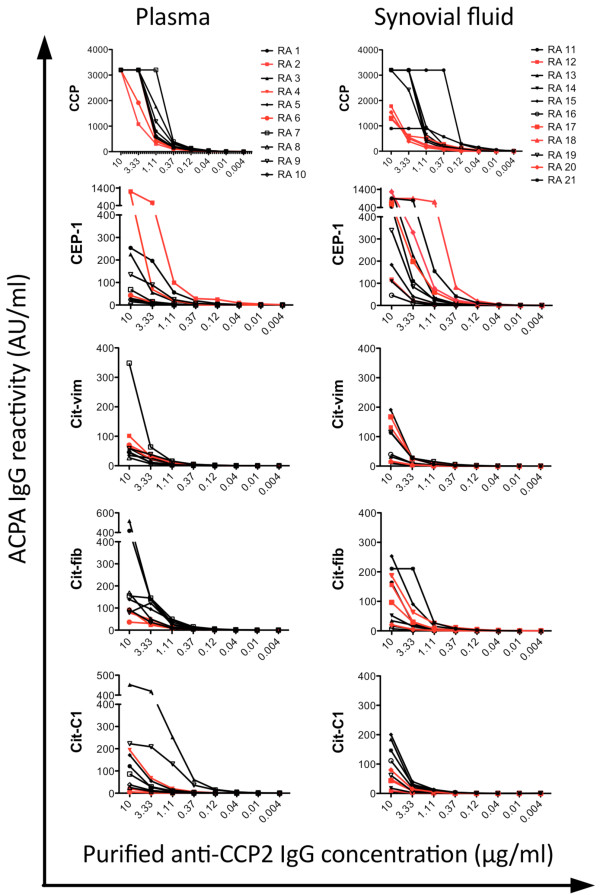
**Anti-CCP2 IgG contains autoantibodies directed to citrullinated proteins/peptides (ACPAs) of various fine specificities.** Analysis was conducted by enzyme-linked immunosorbent assay (ELISA). ACPA responses in purified anti-CCP2 IgG eluates were measured by ELISA in 10 plasma samples (left panel) and 11 synovial fluid samples (right panel). Anti-CCP2 IgG response (top panel), anti-CEP-1 IgG response, anti-Cit-vim IgG response, anti-Cit-fib IgG response, and anti-Cit-C1 IgG response (bottom panel) are shown. X-axis shows anti-CCP2 IgG eluate concentrations (μg/mL). Y-axis shows ACPA levels (AU/mL). Anti-CCP2 IgG eluates highlighted in red indicate samples with particularly weak binding to the CCP2 ELISA plates. CCP, cyclic citrullinated peptide; CEP-1, citrullinated α-enolase peptide 1 (amino acid 5-21); Cit-C1, citrullinated triple helical peptide/epitope on collagen type II (amino acid 359-369); Cit-fib, citrullinated fibrinogen peptide (β-chain, amino acid 36-52); Cit-vim, citrullinated vimentin peptide (amino acid 60-75); IgG, immunoglobulin G.

Anti-CCP2 reactivity was monitored throughout the purification process by using the Immunoscan CCPlus® ELISA. No anti-CCP2 IgG response could be detected in any of the Protein G or CCP2 column flow-through fractions, whereas strong CCP2 reactivity was recorded in all Protein G column eluate fractions and in all CCP2 column eluate fractions (data not shown). The purified anti-CCP2 IgG eluate fractions as well as the corresponding flow-through fractions from 10 plasma and 11 SF samples were further tested (by ELISA) in serial dilutions for reactivity with CCP2 as well as four well-characterized ACPA epitopes on citrullinated α-enolase (CEP-1), vimentin (Cit-vim), fibrinogen (Cit-fib), and collagen type II (Cit-C1). Differences in binding capacity to CCP2 were observed between anti-CCP2 IgG purified from different patients, as demonstrated by marked differences in absorption (that is, AU/mL values) between samples, despite being analyzed at the same antibody concentration (Figure 2). A large majority of CCP2 column eluate fractions from both plasma and SF was positive for all four ACPA fine specificities (Table 2 and Figure 2), whereas the flow-through fractions were consistently negative (data not shown).

**Table 2:** **ACPA IgG responses (AU/mL) in 10 plasma and 11 synovial fluid samples “pre” and “post” CCP2 affinity column purification**

Plasma	CCP		CEP-1		Cit-vim		Cit-fib		Cit-C1	
	Pre	Post	Pre	Post	Pre	Post	Pre	Post	Pre	Post
RA 1	>3,200	>3,200	142	254	<10	45	17	417	17	122
RA 2	505	>3,200	104	1,207	<10	101	18	92	<10	20
RA 3	>3,200	>3,200	44	225	18	59	101	518	333	333
RA 4	>3,200	>3,200	97	1,052	30	65	33	79	77	195
RA 5	>3,200	>3,200	24	30	21	35	104	139	114	172
RA 6	>3,200	>3,200	13	43	30	70	18	36	<10	<10
RA 7	>3,200	>3,200	140	69	107	348	282	155	170	86
RA 8	>3,200	>3,200	11	15	<10	27	42	168	17	26
RA 9	763	>3,200	17	135	<10	59	<10	79	73	222
RA 10	1,151	>3,200	<10	24	<10	47	<10	92	18	38
Median	3,200	3,200	34	102	9	59	25.5	115.5	45.5	104
**Synovial fluid**	**CCP**		**CEP-1**		**Cit-vim**		**Cit-fib**		**Cit-C1**	
	**Pre**	**Post**	**Pre**	**Post**	**Pre**	**Post**	**Pre**	**Post**	**Pre**	**Post**
RA 11	2,447	>3,200	173	447	31	14	28	163	<10	<10
RA 12	311	1,780	<10	117	15	131	<10	156	<10	<10
RA 13	596	>3,200	15	1,339	19	32	<10	34	<10	185
RA 14	1,196	>3,200	22	109	<10	<10	<10	152	30	18
RA 15	508	>3,200	67	183	<10	191	<10	253	<10	200
RA 16	435	>3,200	16	46	<10	40	<10	<10	<10	111
RA 17	378	1,311	51	610	<10	167	<10	97	<10	44
RA 18	592	1,282	688	900	<10	17	<10	17	<10	48
RA 19	1,084	>3,200	85	339	23	115	17	187	19	62
RA 20	515	1,537	67	1,271	<10	17	<10	21	<10	80
RA 21	566	897	101	900	<10	113	<10	80	<10	146
Median	566	3,200	67^a^	447^a^	0	40	0	97	0	62

“Pre” samples were tested at a dilution of 1:100, and “Post” samples were tested at 10 μg/mL. Cutoffs for positivity: 25 AU/mL (CCP) and 10 AU/mL (CEP-1, Cit-vim, Cit-fib, and Cit-C1). ^a^Anti-CEP-1 IgG levels in synovial fluid were significantly higher than the other anti-citrullinated protein/peptide antibody (ACPA) levels (*P* <0.01). CCP, cyclic citrullinated peptide; CEP-1, citrullinated α-enolase peptide 1 (amino acid 5-21); Cit-C1, citrullinated triple helical peptide/epitope on collagen type II (amino acid 359-369); Cit-fib, citrullinated fibrinogen peptide (β-chain, amino acid 36-52); Cit-vim, citrullinated vimentin peptide (amino acid 60-75); IgG, immunoglobulin G; RA, rheumatoid arthritis.

Five plasma samples and 10 SF samples were considered negative for one or several ACPA fine specificities on the basis of the cutoff value for positivity of 10 AU/mL. Still, purified anti-CCP2 IgGs from these samples were considered positive (that is, above the cutoff value for positivity) (Table 2). The reason for this discrepancy is most likely due to differences in ACPA concentrations between the purified anti-CCP2 IgG eluates, which were tested at 10 μg/mL, and the original “pre” plasma and SF samples, which were tested at a lower concentration (that is, a dilution of 1:100).

No correlations could be observed between strong CCP2-binding and presence of specific ACPA fine-specificity patterns. In fact, several anti-CCP2 IgG eluates with weak binding to CCP2 seemed to have particularly strong binding to CEP-1 (Figure 2). Moreover, purified anti-CCP2 IgG from SF showed significantly stronger reactivity with CEP-1 compared with the other citrullinated peptides (*P* <0.01). Median reactivities were 447 AU/mL for anti-CEP-1 and 40, 97, and 62 AU/mL for anti-Cit-vim, anti-Cit-fib, and anti-Cit-C1, respectively (Table 2). This observation was mirrored by significantly higher anti-CEP-1 IgG levels in the original “pre” SF samples compared with the other ACPA fine specificities. This anti-CEP-1 “enrichment” was specific for SF and could not be seen in plasma/anti-CCP2 IgG purified from plasma.

**Figure 3:**
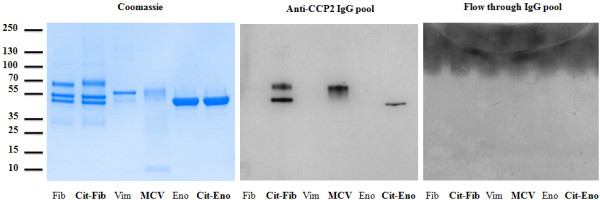
**Anti-CCP2 IgG contains autoantibodies directed to citrullinated proteins/peptides (ACPAs) of various fine specificities.** Analysis was conducted by sodium dodecyl sulphate-polyacrylamide gel electrophoresis (SDS-PAGE) and Western blot. SDS-PAGE was followed by Coomassie staining and Western blot of uncitrullinated and citrullinated: fibrinogen, mutated vimentin, and α-enolase (all at 1 μg/well). Coomassie staining (left), Western blot using a pool of 11 purified anti-CCP2 IgG eluate fractions from synovial fluid (center) or a pool of the corresponding CCP2 column IgG flow-through fractions (right) are shown. CCP, cyclic citrullinated peptide; IgG, immunoglobulin G.

Reactivity of the purified ACPA was further analyzed by Western blot, in which a pool of 11 purified anti-CCP2 IgG eluate fractions from SF bound *in vitro*-citrullinated, but not native human fibrinogen α- and β-chains, α-enolase, and mutated vimentin, whereas the corresponding CCP2 column flow-through IgG pool bound neither the citrullinated nor the native proteins (Figure 3).

### Purified anti-CCP2 IgGs bind antigen targets in RA synovial tissue and SF cells

**Figure 4:**
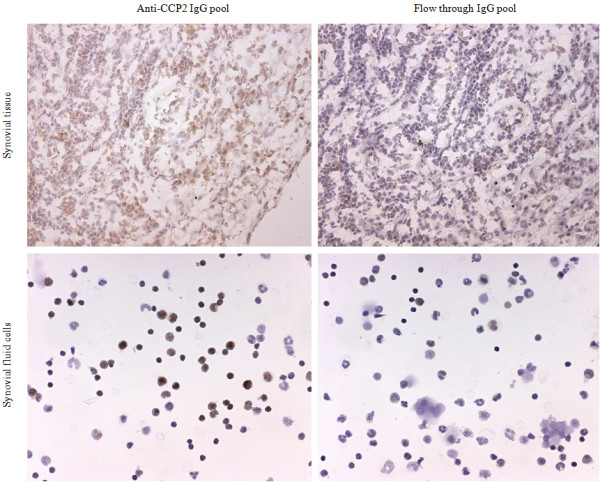
**Purified anti-CCP2 IgGs bind** ***in vivo*** **-generated epitopes in rheumatoid arthritis (RA) synovial tissue and synovial fluid (SF) cells.** Immunohisto-/immunocytochemical stainings of RA synovial tissue (top panel) and RA synovial fluid cells (bottom panel) using a pool of 26 purified anti-CCP2 IgG eluate fractions from SF (left panel) or a pool of the corresponding CCP2 column IgG flow-through fractions (right panel). Magnifications: 10× (synovial tissue) and 25× (SF cells). Representative pictures from n = 3 RA patients (synovial tissue) and n = 3 RA patients (SF cells) are shown. CCP, cyclic citrullinated peptide; IgG, immunoglobulin G.

A pool of 26 purified anti-CCP2 IgG eluate fractions from SF and the corresponding flow-through IgG pool were biotinylated and tested for the ability to bind *in vivo*-generated citrullinated proteins by immunohisto-/immunocytochemistry (Figure 4). Infiltrating cells in inflamed RA synovial tissue stained positive using the ACPA IgG pool, whereas staining with the corresponding flow-through IgG pool was negative. Likewise, RA SF cells stained positive with the ACPA pool but negative with the flow-through pool.

## Discussion

Using an efficient and robust method to affinity-purify ACPAs, we were able to determine the concentration and the proportion of CCP2-reactive IgG molecules in plasma and SF of patients with RA. Based on the samples analyzed in our study, approximately 2% of the IgG pool in both plasma and SF are CCP2-reactive, which corresponds to anti-CCP2 IgG concentrations of 0.2 and 0.06 mg/mL (median values) in plasma and SF, respectively. These concentrations are somewhat higher than what was reported in another study [22] but are in line with what has been described more recently [29] and in studies of other antigen-specific IgG responses [31, 32]. In SF samples from four patients, more than 6% of the IgG molecules were CCP2-reactive. This high proportion of ACPA may suggest a local production or enrichment (or both) of autoantibodies in the inflamed joint, which also has been proposed in a previous study, in which higher ACPA levels were detected in SF, compared to serum, after normalization against total IgG [7]. This observation is supported by a recent study, in which the ACPA-specific memory B cells were estimated to constitute as much as 30% of the total memory B-cell pool in RA SF [33].

Our ELISA data formally demonstrate that the CCP2 peptides commonly used in diagnostic ELISAs *de facto* act as surrogate antigens for the four most extensively studied ACPA fine specificities in RA: anti-CEP-1, anti-Cit-vim, anti-Cit-fib, and anti-Cit-C1 antibodies. The finding that purified anti-CCP2 IgG identifies citrullinated fibrinogen, α-enolase, and mutated vimentin by immunoblotting confirms and extends data previously published by Ioan-Facsinay and colleagues, who could show that antibodies eluted from CCP2 ELISA plates bound to citrullinated fibrinogen [22].

The differences seen in binding to CCP2 between the different anti-CCP2 IgG eluates when analyzed by using the CCPlus® ELISA suggest that differences in affinity for the CCP2 peptides exist between patients, which in turn may depend on the composition of ACPA fine specificities in the different samples. However, we have not been able to identify a specific ACPA profile corresponding to strong CCP2 binding. We observed, on the other hand, that several anti-CCP2 IgG eluates with weak binding to CCP2 showed particularly strong binding to CEP-1, which may suggest that anti-CEP-1 antibodies have lower affinity for the CCP2 peptides compared with the other ACPA fine specificities investigated here. Still, it is important to note that all anti-CEP-1 antibodies were captured by the CCP2 column, as no CEP-1 reactivity was detected in the flow-through fractions.

In SF and in anti-CCP2 IgG purified from SF, reactivity with the CEP-1 peptide was significantly stronger than reactivity with the other citrullinated peptides. These data are in line with those of a recently published study by Amara and colleagues, demonstrating that CEP-1 reactive B-cell clones were more commonly isolated from RA SF than cit-vim and cit-fib reactive B-cell clones [33]. The results in both their study and our study may be influenced by how well the different peptide ELISAs perform. Still, such differences were not seen in plasma or in anti-CCP2 IgG purified from plasma. Hence, the data suggest a specific enrichment or production (or both) of anti-CEP-1 antibodies in the joint.

## Conclusions

Our study demonstrates that anti-CCP2 reactive antibodies constitute at least four different RA-associated ACPA fine specificities and furthermore that anti-CCP2 IgGs bind *in vivo*-generated antigens in the rheumatoid joint. Isolation of human ACPA by using affinity columns with conjugated citrullinated antigens, such as the CCP2 column described here, or the mutated citrullinated vimentin column, previously described by Harre and colleagues [34], or the more recently described purification of ACPA reactive with various citrullinated CII epitopes, which were shown to bind rheumatoid cartilage [35], will allow important functional and structural *in vitro* and *in vivo* studies aimed at elucidating the mode of action and pathogenic potential of human ACPA.

## Abbreviations

ACPA: anti-citrullinated protein/peptide antibody
CCP: cyclic citrullinated peptide
CEP-1: citrullinated α-enolase peptide 1 (amino acid 5-21)
CII: collagen type II
Cit-C1: citrullinated triple helical peptide/epitope on collagen type II (amino acid 359-369)
Cit-fib: citrullinated fibrinogen peptide (β-chain, amino acid 36-52)
Cit-vim: citrullinated vimentin peptide (amino acid 60-75)
DTT: dithiothreitol
ECL: enhanced chemiluminescence
ELISA: enzyme-linked immunosorbent assay
HRP: horseradish peroxidase
IgG: immunoglobulin G
LDS: lithium dodecyl sulphate
PAD2: peptidylarginine deiminase 2
PBS: phosphate-buffered saline
RA: rheumatoid arthritis
SDS-PAGE: sodium dodecyl sulphate-polyacrylamide gel electrophoresis
SF: synovial fluid.
